# Epidemiologic Trends and Factors Associated With Overall Survival for Patients With Hepatobiliary Neuroendocrine Neoplasms in the United States

**DOI:** 10.1002/cnr2.70410

**Published:** 2025-11-28

**Authors:** Xinlei Zhou, Chenxi Zhou, Yi Lu, Shenghao Lin, Che Fu, Mengfan Wei, Leitao Sun, Kaibo Guo

**Affiliations:** ^1^ The Third Clinical Medical School, the Rehabilitation Medical School Zhejiang Chinese Medical University Hangzhou China; ^2^ The First Affiliated Hospital of Zhejiang Chinese Medical University (Zhejiang Provincial Hospital of Chinese Medicine) Hangzhou China; ^3^ School of Medical Technology and Information Engineering Zhejiang Chinese Medical University Hangzhou China; ^4^ Department of Oncology Hangzhou First People's Hospital, Westlake University School of Medicine Hangzhou China

## Abstract

**Background:**

Neuroendocrine neoplasms (NENs) constitute a heterogeneous group of rare tumors that most commonly originate from the gastrointestinal tract and lungs, with a consistently rising global incidence over recent decades. Hepatobiliary neuroendocrine neoplasms (HB‐NENs), defined as primary NENs arising in the liver and biliary tract, are exceedingly uncommon, accounting for less than 0.5% of all NENs. Owing to their low incidence, atypical clinical presentations, and the paucity of large‐scale datasets, no distinct World Health Organization (WHO) classification criteria have yet been established for these entities. Epidemiological characteristics and prognostic studies of HB‐NENs remain extremely limited. Current prognostic evaluation relies predominantly on the American Joint Committee on Cancer (AJCC) and European Neuroendocrine Tumor Society (ENETS) TNM staging systems, whereas population‐level investigations into incidence trends, survival determinants, and individualized predictive tools for HB‐NENs are still largely absent.

**Aims:**

To investigate epidemiological trends (1992–2020) and prognostic factors associated with overall survival in a nationally representative cohort of patients with hepatobiliary neuroendocrine neoplasms using the Surveillance, Epidemiology, and End Results registry, with subgroup stratification by sex and race. Additionally, we aim to develop a prognostic nomogram integrating significant predictors to quantify individualized survival probabilities for this population.

**Methods:**

In this cohort study, patients diagnosed with hepatobiliary neuroendocrine neoplasms between January 1, 1975, and December 31, 2020, were identified from the Surveillance, Epidemiology, and End Results Program. Related data were used for epidemiologic and survival analysis, as well as the development and validation of a nomogram to predict the overall survival probability of individual hepatobiliary neuroendocrine neoplasms patients.

**Results:**

The age‐adjusted incidence rate of hepatobiliary neuroendocrine neoplasms increased 1.76‐fold from 1992 to 2020 (annual percentage change [APC], 2.73; 95% CI, 1.86−3.65; *p* < 0.05). Furthermore, the incidence of hepatobiliary neuroendocrine neoplasms in the extrahepatic bile duct increased most significantly (APC, 3.73; 95% CI, 2.03−5.46; *p* < 0.05), and the gallbladder also increased most significantly (APC, 3.51; 95% CI, 1.97−5.08; *p* < 0.05), whereas patients with hepatobiliary neuroendocrine neoplasms in the liver had no significant difference. As for tumor type, the incidence increased in hepatobiliary neuroendocrine carcinomas (APC, 2.93; 95% CI, 1.34−4.53; *p* < 0.05) but the incidence was stable in hepatobiliary neuroendocrine tumors. On multivariate analyzes, age at diagnosis, race, tumor size, grade, tumor type, tumor stage, and surgery were significantly associated with overall survival for hepatobiliary neuroendocrine neoplasms patients. Furthermore, a nomogram based on significant associated factors (age at diagnosis, race, tumor size, stage, type, grade, chemotherapy and surgery) was constructed to predict the 6‐month, 1‐year and 2‐year survival probability, with the concordance indexes of 0.829 (95% CI, 0.800−0.857) for the training cohort and 0.801 (95% CI, 0.745−0.857) for the validation cohort.

**Conclusion:**

In this study, the incidence of hepatobiliary neuroendocrine neoplasms has exhibited a persistent upward trend over nearly two decades. Furthermore, this study proposes that a nomogram comprising eight prognostic parameters can effectively quantify the mortality risk in hepatobiliary neuroendocrine neoplasms patients.

## 1 Introduction

Neuroendocrine neoplasms (NENs)are a heterogeneous group of rare tumors that most commonly arise in the gastrointestinal tract and lungs, with an increasing global incidence over recent decades [1]. According to the World Health Organization (WHO), NENs are classified into four grades—neuroendocrine tumor (NET) G1, NET G2, NET G3, and NEC—based on mitotic count and Ki‐67 proliferation index [2, 3]. While NENs most frequently metastasize to the liver, primary neuroendocrine neoplasms of the liver and biliary tract—collectively termed hepatobiliary NENs (HB‐NENs)—are exceedingly rare, accounting for less than 0.5% of all NENs [4, 5]. Owing to their infrequency, atypical clinical presentations, and limited comprehensive datasets, WHO‐NEN has not furnished distinct classification criteria for these tumors.

Presently, a paucity of data exists concerning the epidemiological attributes and survival analysis of HB‐NEN patients. Prognostic evaluation for HB‐NENs is predominantly reliant upon the American Joint Committee on Cancer and European Neuroendocrine Tumor TNM staging systems. Furthermore, supplementary clinical and pathological attributes, encompassing age, gender, ethnicity, tumor grade, and tumor location, may also hold significance in determining patients' prognosis. Given the scarcity of research on these uncommon neoplasms, acquiring a substantial corpus of definitive data regarding etiology, pathogenesis, standard therapeutic modalities, and prognosis through multicenter large‐scale investigations proves to be a formidable challenge. Consequently, few population‐level studies have systematically examined incidence trends or survival determinants specific to HB‐NENs. To address this gap, this study conducted a population‐based study using the Surveillance, Epidemiology, and End Results (SEER) database to evaluate the epidemiological and prognostic features of HB‐NENs. Furthermore, we developed and validated a prognostic nomogram incorporating key clinical variables to predict 6‐month, 1‐year, and 2‐year overall survival (OS) in HB‐NEN patients.

## 2 Methods

### 2.1 Data Source

For this investigation, we harnessed the formidable potential of the SEER database, an authoritative fount of knowledge regarding cancer epidemiology, encompassing incidence and prevalence, as well as clinical attributes including primary tumor site, tumor morphological characteristics, stage at diagnosis, initial treatment regimen, and vital status monitoring, all within the United States. To identify patients who were diagnosed with HB‐NENs from January 1, 1975, to December 31, 2020, we utilized histologic codes sourced from the WHO Classification of Tumours of the Digestive System, Fifth Edition [6], in conjunction with site codes (as detailed in Table S1). In accordance with established guidelines, this study adhered to the Strengthening the Reporting of Observational Studies in Epidemiology (STROBE) reporting framework. The SEER database is an anonymous and accessible database from the United States, so ethics approval and written informed consent are not required.

### 2.2 Stage and Classification of HB‐NENs

Owing to the absence of a standardized and pragmatic staging system tailored for HB‐NENs, our research adopted the SEER staging system. In broad terms, tumors were categorized as localized, regional, or distant diseases. Localized HB‐NENs were characterized as malignancies displaying an aggressive nature while remaining entirely confined to the organ of origin. Regional HB‐NENs were demarcated as tumors that exhibited one or more of the following characteristics: (1) extension beyond the confines of the originating organ, encroaching upon adjacent organs or tissues, (2) dissemination into regional lymph nodes through the lymphatic system, or (3) a combination of both extension and involvement of regional lymph nodes. In contrast, distant HB‐NENs were delineated as tumors that had disseminated to anatomical regions remote from the primary tumor site or distant from its origin. In terms of tumor classification, the SEER classification scheme methodically stratifies cases into four grades: Grade 1 (G1), characterized by a high level of differentiation; G2, indicating moderate differentiation; G3, signifying poor differentiation; and G4, representing undifferentiated or anaplastic tumors. However, due to constraints imposed by the limited sample size, we condensed the “grade” variable into two categories: G1–2 (comprising G1 and G2) and G3–4 (encompassing G3 and G4).

### 2.3 Nomogram Construction and Validation

All eligible patients, were subjected to a random division into training and validation groups, with a ratio of 7:3. Multivariate Cox proportional hazards regression models were subsequently deployed to assess factors that correlate with overall survival (OS). The validation of the nomogram primarily hinges on internal assessment within the training cohort and external assessment within the validation cohort, gauged through discrimination and calibration metrics. The principal indicator for evaluating the nomogram's discriminative efficacy is the consistency index (C index), which primarily quantifies disparities between the projected and actual outcomes. The calibration curve was employed to compare survival prognostications generated by the nomogram with the real observed survival rates. Moreover, to ascertain the clinical utility of the nomogram for predicting 6‐month, 1‐year, and 2‐year survival, decision curve analysis (DCA) was undertaken.

### 2.4 Statistical Analysis

The statistical analysis was executed within the timeframe of May 1 to July 31, 2023. Descriptive statistics, *t*‐tests, or *χ* ^2^ tests were employed to analyze patients' fundamental clinical attributes, including the year of diagnosis, age at diagnosis, race, gender, marital status, tumor site, tumor size, grade, tumor type, tumor stage, treatment modalities (radiation therapy, chemotherapy, surgery), follow‐up time, and survival status.

The primary outcome of interest revolved around cancer‐specific mortality, extracted from the variable known as “cause‐specific death classification”. The SEER*Stat program derives estimates for survival duration by calculating the time interval between the date of diagnosis and the date of the last recorded follow‐up, which corresponds to the study's cutoff point. The age‐adjusted incidence rates from “Incidence—SEER Research Data, 8 Registries, Nov 2020 Sub (1975–2020)” and “Incidence—SEER Research Data, 12 Registries, Nov 2020 Sub (1992–2020)”, were computed utilizing SEER*Stat software, version 8.4.1.2, which is developed by the Surveillance Research Program at the National Cancer Institute. To calculate the annual percentage change (APC), a simple linear model was employed. Initially, the logarithm of the yearly age‐adjusted rates was regressed against time, and subsequently, a transformation of the slope was applied to derive the percentage change per year [7]. The APCs were standardized across scales, facilitating the ability to make meaningful comparisons of incidence rates between cohorts, whether they involve rare or common malignant neoplasms [8].

The primary outcome of interest revolved around cancer‐specific mortality, extracted from the variable known as “cause‐specific death classification”. The SEER*Stat program derives estimates for survival duration by calculating the time interval between the date of diagnosis and the date of the last recorded follow‐up, which corresponds to the study's cutoff point. The study cutoff date was December 31, 2020.

We employed Cox proportional hazards multivariate regression to assess the correlation between various factors, including age at diagnosis, race, gender, marital status, tumor site, tumor size, grade, tumor type, tumor stage, and treatment modalities (radiation, chemotherapy, surgery), with overall survival (OS). This analysis involved calculating hazard ratios (HRs) and their corresponding 95% confidence intervals (CIs), with adjustments made for other factors. Statistical analyzes were executed using R software, version 3.6.1. All *p*‐values were obtained from two‐sided tests, and outcomes were regarded as statistically significant when *p* < 0.05.

## 3 Results

### 3.1 Patient Characteristics

Between 1975 and 2020, a comprehensive cohort comprising 390 patients diagnosed with HB‐NENs was identified within the SEER database. These patients exhibited a mean age at diagnosis of 61 years (standard deviation [SD] of 15), with a median age of 63 years (ranging from 21 to 90 years). The screening process is illustrated in Figure S1. Among the eligible 390 patients, 274 were randomly assigned to the training cohort, while the remaining 116 were allocated to the validation cohort. The gender distribution approached parity, with 178 (45.6%) male and 212 (54.4%) female patients, as detailed in Table 1. Demographically, 70.8% (276) of the patients were of White ethnicity, 13.1% (51) were of Black ethnicity, and 16.2% (63) belonged to other racial categories. Among the 216 HB‐NENs (55.4%) for which the grade was known, 80 (20.5%) were categorized as G1, 34 (8.7%) as G2, 72 (18.5%) as G3, and 30 (7.7%) as G4. At the time of diagnosis, 149 (38.2%) patients had localized disease, 128 (32.8%) presented with regional disease, and 113 (28.9%) were diagnosed with distant disease. Regarding the primary tumor sites of HB‐NENs, the gallbladder and extrahepatic bile duct were the two most frequently affected sites (139 cases each, 35.6%), followed by the liver (111 cases, 28.4%). Baseline characteristics of the training and validation cohorts are summarized in Table 1. *p* values were presented for descriptive comparison only, and no clinically meaningful differences were observed between cohorts.

**TABLE 1:** Clinicopathological characteristics of patients with hepatobiliary neuroendocrine neoplasm (HB‐NEN).

	Overall	Training cohort	Validation cohort	*p*
Characteristics	390	274	116	
Year of diagnosis (%)				0.092
before 2005	134 (34.4)	85 (31.0)	49 (42.2)	
2006–2010	66 (16.9)	53 (19.3)	13 (11.2)	
2011–2015	81 (20.8)	57 (20.8)	24 (20.7)	
2016–2020	109 (27.9)	79 (28.8)	30 (25.9)	
Age at diagnosis (%)				0.167
< 60 years	168 (43.1)	119 (43.4)	49 (42.2)	
60–74 year	142 (36.4)	93 (33.9)	49 (42.2)	
≥ 75 year	80 (20.5)	62 (22.6)	18 (15.5)	
Race (%)				0.883
White	276 (70.8)	194 (70.8)	82 (70.7)	
Black	51 (13.1)	37 (13.5)	14 (12.1)	
Other	63 (16.2)	43 (15.7)	20 (17.2)	
Sex (%)				0.152
Female	212 (54.4)	142 (51.8)	70 (60.3)	
Male	178 (45.6)	132 (48.2)	46 (39.7)	
Marital status (%)				0.663
Married	217 (55.6)	150 (54.7)	67 (57.8)	
Unmarried	173 (44.4)	124 (45.3)	49 (42.2)	
Tumor site (%)				0.953
Liver	111 (28.7)	78 (28.5)	34 (29.3)	
Gallbladder	139 (35.6)	97 (35.4)	42 (36.2)	
Extrahepatic bile duct	139 (35.6)	99 (36.1)	40 (34.5)	
Tumor size (%)				0.537
≤ 20 mm	95 (24.4)	70 (25.5)	25 (21.6)	
21–50 mm	54 (13.8)	41 (15.0)	13 (11.2)	
> 50 mm	63 (16.2)	43 (15.7)	20 (17.2)	
Unknown	178 (45.6)	120 (43.8)	58 (50.0)	
Tumor grade (%)				0.206
G1–2	114 (29.2)	87 (31.8)	27 (23.3)	
G3–4	102 (26.2)	67 (24.5)	35 (30.2)	
Unknown	174 (44.6)	120 (43.8)	54 (46.6)	
Tumor type (%)				0.332
NEC	228 (58.5)	165 (60.2)	63 (54.3)	
NET	162 (41.5)	109 (39.8)	53 (45.7)	
Tumor stage (%)				0.816
Distant	113 (29.0)	77 (28.1)	36 (31.0)	
Localized	149 (38.2)	105 (38.3)	44 (37.9)	
Regional	128 (32.8)	92 (33.6)	36 (31.0)	
Radiation therapy (%)				1.000
No	356 (91.3)	250 (91.2)	106 (91.4)	
Yes	34 (8.7)	24 (8.8)	10 (8.6)	
Chemotherapy (%)				0.809
No	264 (67.7)	187 (68.2)	77 (66.4)	
Yes	126 (32.3)	87 (31.8)	39 (33.6)	
Surgery (%)				0.845
No	166 (42.6)	118 (43.1)	48 (41.4)	
Yes	224 (57.4)	156 (56.9)	68 (58.6)	
Follow‐up time (months)	20 [6, 68]	19.5 [6, 61]	21.5 [8, 88.5]	0.121
Survival status (%)				0.913
Alive	148 (37.9)	103 (37.6)	45 (38.8)	
Death	242 (62.1)	171 (62.4)	71 (61.2)	

*Note: p* values were provided for descriptive comparison of baseline characteristics between training and validation cohorts.

### 3.2 Annual Incidence

**FIGURE 1:**
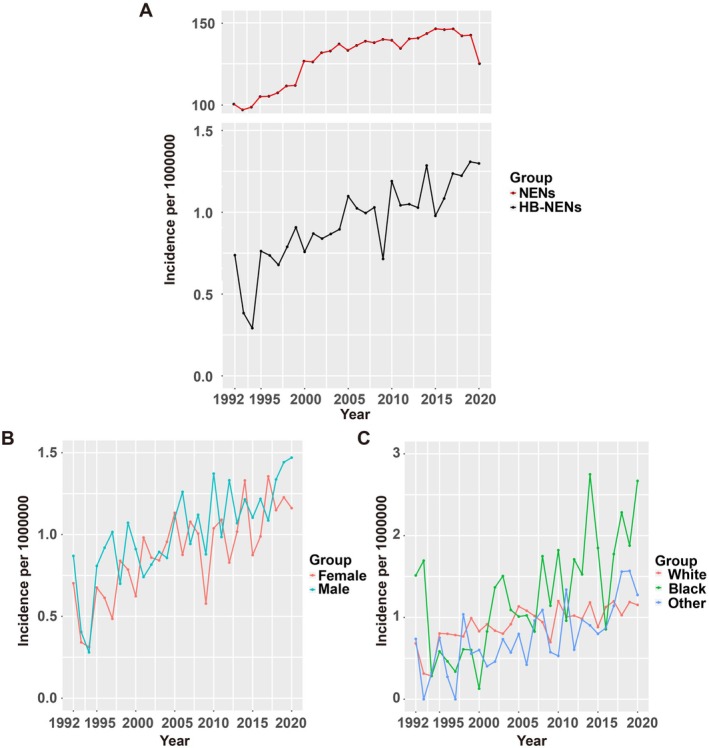
Incidence of Hepatobiliary Neuroendocrine Neoplasms (HB‐NENs) Over Time by Sex and Race. (A) Annual age‐adjusted incidence of all HB‐NENs by year (1975–2020). The incidence is presented as the number of tumors per 1000 000 population (with 95% CIs presented as whiskers) age‐adjusted for the 2000 US standard population. (B) Time‐trend analyzes of the incidence of HB‐NENs by sex (1975–2020). (C) The incidence of HB‐NENs by race (1975–2020).

Utilizing population data extracted from the SEER program, we computed the age‐adjusted incidence of HB‐NENs per 1 000 000 individuals annually, referring to the 2000 US standard population. In 1992, the overall age‐adjusted incidence of HB‐NENs stood at 0.74 per 1 000 000 individuals, and this figure rose to 1.30 per 1 000 000 individuals by the year 2020 (as documented in Table S2). This increase was associated with an annual percentage change (APC) of 2.73 (with a 95% CI ranging from 1.83 to 3.65), as depicted in Figure 1A. This contrasts with the annual age‐adjusted incidence of all malignant neoplasms, which exhibited an APC of 0.82 (with a 95% CI spanning from 0.59 to 1.09). In terms of gender, the age‐adjusted incidence rates for female patients with HB‐NENs displayed a significant upward trend over time, characterized by an APC of 3.30 (with a 95% CI ranging from 2.22 to 4.39). In contrast, male patients exhibited a more modest APC of 2.03 (with a 95% CI from 0.87 to 3.20), as illustrated in Figure 1B.

Incidence rates among people other than white populations increased until 2020 (White populations: APC, 2.01 [95% CI, 0.88–3.15]; Black populations: APC, 4.59 [95% CI, 1.97–7.28]; other populations: APC, 4.78 [95% CI, 2.55–7.06]) (Figure 1C). As for the tumor site (Figure S2A) of HB‐NENs, incidence rate of liver was stable (APC, 1.14 [95% CI, −0.13 to 2.42], Table S3), while incidence rates of gallbladder (APC, 3.51 [95% CI, 1.97–5.08], Table S4) and extrahepatic bile duct (APC, 3.73 [95% CI, 2.03–5.46], Table S5) increased. As shown in Figure S2B, incidence rates of HB‐NECs increased during the period from 1992 to 2020 (APC, 2.93 [95% CI, 1.34–4.53], Table S6), whereas rates for HB‐NETs (APC, 2.12 [95% CI, 0.80–3.45], Table S7) remained unchanged.

### 3.3 Multivariate Analysis of OS

**FIGURE 2:**
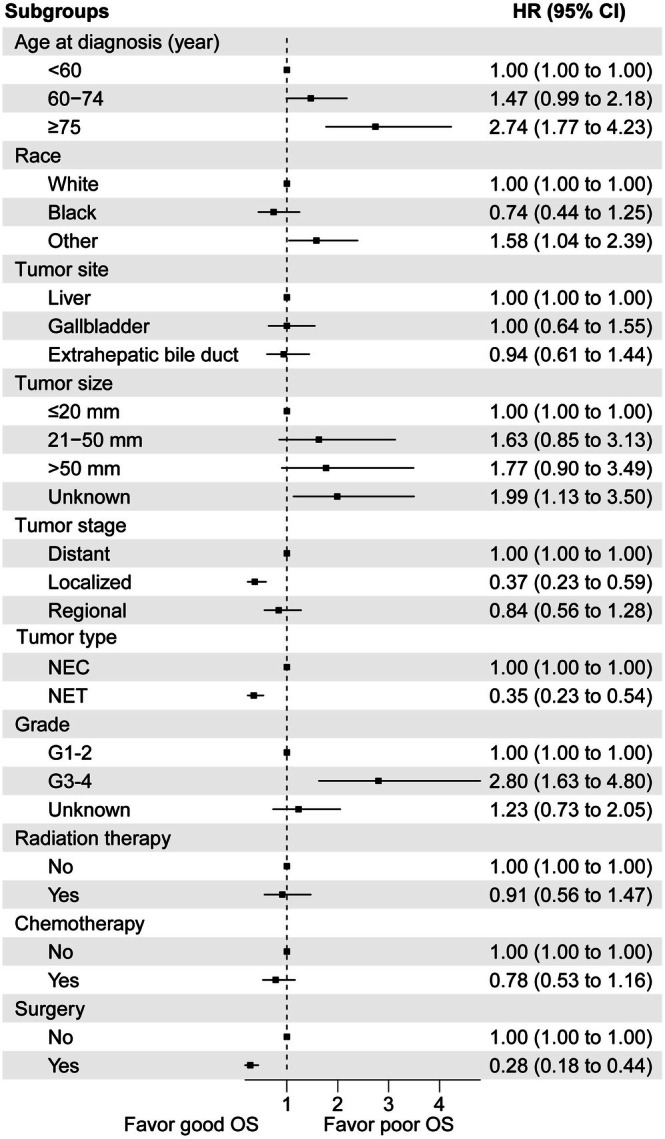
Multivariate regression analysis for hepatobiliary neuroendocrine neoplasms (HB‐NENs). HR indicates hazard ratio.

Subsequently, we conducted both univariate and multivariate analyzes to delve deeper into the independent prognostic risk factors for HB‐NET patients, employing the Cox proportional hazards regression model. In this model, we incorporated potentially prognostic parameters, encompassing age, race, gender, marital status, primary site, tumor size, tumor grade, tumor type, tumor stage, as well as the receipt of radiation treatment, chemotherapy, and surgery. With the exception of marital status, all other parameters, namely age, race, gender, primary site, tumor size, tumor grade, tumor type, tumor stage, radiation treatment, chemotherapy, and surgery, exhibited significant associations with survival, as delineated in Table 2 and visually represented in Figure 2.

**TABLE 2:** COX regression analysis of the prognostic factors for OS in patients with hepatobiliary neuroendocrine neoplasm (HB‐NEN).

Characteristics	Univariate		Multivariate	
	HR (95% CI)	*p*	HR (95% CI)	*p*
Age at diagnosis				
< 60 years	Reference		Reference	
60–74 year	1.83 (1.26–2.66)	0.002	1.47 (0.99–2.18)	0.057
≥ 75 year	3.83 (2.62–5.61)	< 0.001	2.74 (1.77–4.23)	< 0.001
Race				
White	Reference		Reference	
Black	0.8 (0.5–1.29)	0.357	0.74 (0.44–1.25)	0.261
Other	1.89 (1.29–2.78)	0.001	1.58 (1.04–2.39)	0.031
Sex				
Female	Reference			
Male	1.16 (0.86–1.57)	0.337		
Marital status				
Married	Reference			
Unmarried	0.89 (0.66–1.21)	0.468		
Tumor site				
Liver	Reference		Reference	
Gallbladder	0.86 (0.60–1.23)	0.411	1 (0.64–1.55)	0.998
Extrahepatic bile duct	0.62 (0.42–0.9)	0.013	0.94 (0.61–1.44)	0.766
Tumor size				
≤ 20 mm	Reference		Reference	
21–50 mm	2.86 (1.72–4.74)	< 0.001	1.63 (0.85–3.13)	0.144
> 51 min	3.39 (2.07–5.56)	< 0.001	1.77 (0.9–3.49)	0.098
Unknown	3.81 (2.52–5.76)	< 0.001	1.99 (1.13–3.5)	0.018
Grade				
G1–2	Reference		Reference	
G3–4	6.76 (4.25–10.76)	< 0.001	2.8 (1.63–4.8)	< 0.001
Unknown	2.93 (1.89–4.53)	< 0.001	1.23 (0.73–2.05)	0.438
Tumor type				
NEC	Reference		Reference	
NET	0.26 (0.18–0.37)	< 0.001	0.35 (0.23–0.54)	< 0.001
Tumor stage				
Distant	Reference		Reference	
Localized	0.21 (0.14–0.31)	< 0.001	0.37 (0.23–0.59)	< 0.001
Regional	0.40 (0.28–0.58)	< 0.001	0.84 (0.56–1.28)	0.425
Radiation therapy				
No	Reference		Reference	
Yes	1.73 (1.10–2.74)	0.019	0.91 (0.56–1.47)	0.704
Chemotherapy				
No	Reference		Reference	
Yes	2.55 (1.86–3.5)	< 0.001	0.78 (0.53–1.16)	0.219
Surgery				
No	Reference		Reference	
Yes	0.21 (0.15–0.28)	< 0.001	0.28 (0.18–0.44)	< 0.001

Compared with age < 60 years, the risk of death for age ≥ 75 years was greatly increased (HR, 2.74; 95% CI, 1.77–4.23). Overall survival was poorer for patients with grade III‐IV (HR, 2.80; 95% CI, 1.63–4.80) after adjustment for other covariates. When we used distant stage as a reference, we observed that patients with localized stage showed a significant difference in better survival (HR, 0.37; 95% CI, 0.23–0.59). Patients with HB‐NETs showed better survival than patients with HB‐NECs (HR, 0.35; 95% CI, 0.23–0.54). By contrast, patients treated with radiation treatment or chemotherapy showed no significant difference in survival.

### 3.4 Nomogram

**FIGURE 3:**
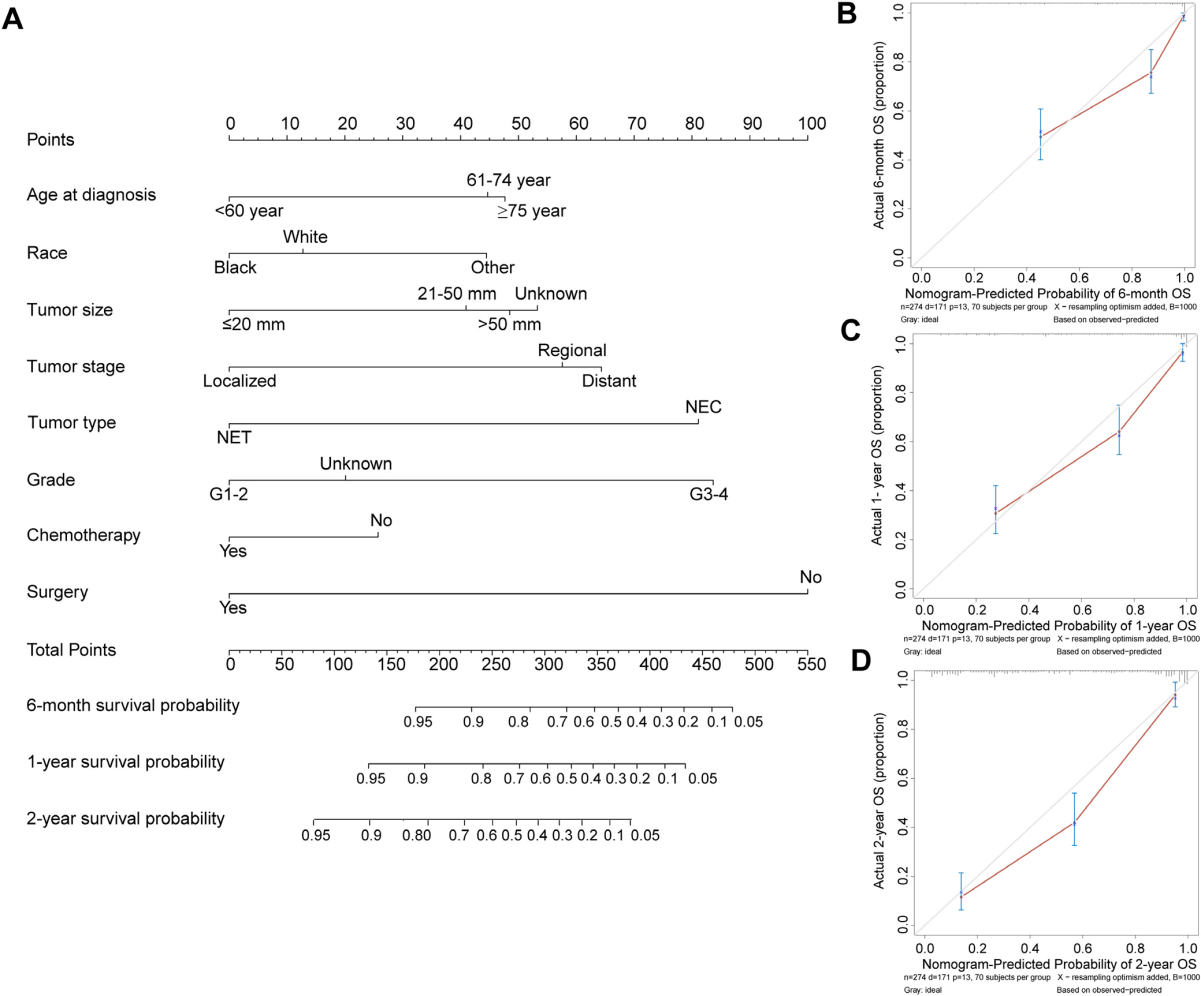
Nomogram to predict the 6‐month, 1‐year and 2‐year survival probabilities of patients with hepatobiliary neuroendocrine neoplasms (HB‐NENs) and the calibration of the nomogram using the training set. (A) Points for age at diagnosis, race, tumor size, tumor stage, tumor type, grade, chemotherapy and surgery are obtained by drawing a line upward from the corresponding values to the “Points” line. The sum of the points of these eight factors is located on the “Total points” line, and a line projected down to the bottom scales determines the probabilities of 6‐month, 1‐year and 2‐year %“overall survival (OS)”. (B) Calibration plots of the nomogram for 6‐month, 1‐year and 2‐year survival %“probabilities in the training set”. The gray line represents the ideal nomogram, and the red line represents the observed nomogram. The predicted probability of OS by the nomogram is projected onto the *x*‐axis, and the actual OS is projected onto the *y*‐axis. Error bars indicate 95% CIs.

Based on the training cohort, a nomogram model was constructed by including factors associated with OS, according to the Cox proportional hazards regression model (Figure 3A). Surgery had the greatest significance, contributing a maximum of 100 points. Tumor type (83 points), grade (80 points), age (78 points), stage (77 points), race (59 points) and tumor size (54 points) were also individually associated with OS (Table S8). Total points of prognostic factors for predicting 6‐month, 1‐year and 2‐year survival probability were shown in Tables S9–S11, respectively. The nomogram was internally and externally validated. In the training cohort and validation cohort, the C indexes for OS prediction in the nomogram were 0.829 (95% CI, 0.800–0.857) and 0.801 (95% CI, 0.745–0.857), respectively. Finally, the calibration plots of the nomogram showed consistency between the nomogram predicted and actual outcomes in the internal (Figure 3B–D) and external (Figure S3A–C) validation, and decision curve analysis showed good clinical benefits in this model (Figure S3D).

## 4 Discussion

In this study, we attempted to analyze the epidemiological, clinical, and prognostic characteristics of hepatobiliary neuroendocrine neoplasms (HB‐NENs) reported up to the year 2020, using data from the SEER database. There were few earlier epidemiological studies to study the incidence trend of HB‐NENs. The overall incidence of HB‐NENs has been notably low, constituting approximately 1% of all NEN cases. However, this incidence has shown a gradual upward trend over the past two decades, particularly among female patients, individuals with tumors located outside the liver, non‐White populations, and those with neuroendocrine carcinomas (NECs). This finding aligns with reports from other national registries, including Germany and Switzerland, where a steady rise in NEN incidence has also been observed over the last two decades, especially among gastrointestinal and pancreatic sites [9, 10]. Moreover, registry refinement efforts in Italy have further clarified site‐specific incidence trends by correcting historical misclassifications in NEN reporting [11]. Female patients with HB‐NENs accounted for a relatively large proportion, more than 50%, which was consistent with previous reports. In the Norwegian national study, females also showed a survival advantage in NENs, further highlighting potential sex‐based biological or diagnostic differences [12]. In a study focusing on primary hepatic neuroendocrine tumors, 291 patients had a median age of 63 years, with 46.4% of them being male [13]. According to case reports, the incidence of biliary NETs was higher in females, accounting for approximately 68% [14]. The rising incidence of HB‐NECs highlights the need for further research to better understand their clinical behavior and inform future treatment approaches.

Previous research has not definitively elucidated the prognostic factors of patients with HB‐NENs. In this study, we conducted a prognostic analysis using the SEER database and confirmed the significance of age at diagnosis, race, tumor stage, tumor type and grade in relation to the prognosis. Our findings are consistent with other studies that HB‐NENs patients with older age, distant stage, NECs or G3–4, appear to have poorer survival outcomes [13, 15, 16]. For primary hepatic or gallbladder neuroendocrine tumors, apart from histological grading, age at diagnosis, unmarried status, low differentiation, and regional/distant disease, were identified as independent risk factors for survival [13, 17, 18, 19]. In terms of race, we found that patients with American Indian/AK Native, Asian/Pacific Islander experienced worse OS compared with White or Black populations. However, no study reported the survival gap among different races and analyzed the reason for the discrepancy This may be partially attributed to limitations in race categorization and socioeconomic adjustment in large registries like SEER [20]. There was no prognostic difference among tumor sites, such as liver, gallbladder and extrahepatic bile duct, further illustrating that HB‐NENs could be studied as a whole, regardless of the different sites.

To better contextualize our SEER‐based findings, we examined relevant population‐based studies from other countries. Although most international registry data report on overall NENs rather than HB‐NENs specifically, these findings offer valuable context regarding diagnostic trends, tumor distribution, and prognostic factors that may also influence HB‐NEN patterns. A Norwegian study (1993–2021) reported a threefold increase in the age‐standardized incidence rate (ASIR) of NENs, from 3.0 to 9.2 per 100 000, primarily due to increased detection of small intestinal and rectal tumors [21]. Similarly, in Taiwan, the ASIR rose from 0.30 to 1.51 per 100 000 between 1996 and 2008 [22], although the overall incidence remained lower than in Western populations. Notably, tumor site distribution varied across regions: rectal and pulmonary NENs were most common in Taiwan, whereas Norway reported higher rates in the small intestine and appendix. These discrepancies may reflect differences in genetic background, diagnostic awareness, screening practices, and healthcare access—factors that may also contribute to the under‐recognition of HB‐NENs globally. Regarding prognosis, the Norwegian registry reported a 5‐year overall survival rate of ~50%, with better outcomes in women and those with localized or well‐differentiated tumors [12]. This aligns with our HB‐NEN findings, where age, tumor stage, grade, and type significantly influenced prognosis. Similar trends have been noted in registries from Germany, Switzerland, and the Italian Itanet registry, which reported increasing incidence, evolving histological subtypes, and the importance of standardized data collection methods [9, 10, 23]. While direct comparisons are limited by differences in tumor sites and inclusion criteria, these international datasets underscore the rising global burden of NENs and suggest that consistent national registry efforts are essential—even for rare subtypes such as HB‐NENs. As we all know, there is no previous study to demonstrate the predictive ability of nomograms for HB‐NENs, and specific and clinically applicable nomograms could accurately estimate the outcome of these patients. Therefore, our nomogram has 8 prognostic parameters (age, race, tumor size, tumor stage, tumor type, grade, chemotherapy and surgery) based on our experience as well as the results of multivariate Cox analysis, which could provide patients with HB‐NENs with simple and accurate prognostic predictions. For example, a 63‐year‐old (29 points), white (22 points) patient with HB‐NECs (83 points) and a 3‐cm tumor (40 points) with poor differentiation (G1; 0 points), localized stage (0 points), treated with surgery (0 points) and without chemotherapy (20 points), would have a 6‐month survival rate of 93% (194 points), a 1‐year survival rate of 88% (194 points), and a 2‐year survival rate of 80% (194 points) according to our nomogram. After verification, this simple and effective tool could more accurately predict survival by evaluating various parameters of HB‐NENs, thereby expediting clinical decision‐making and promoting effective communication with patients and their families.

## 5 Limitation

Our study exhibits several limitations. First, it is worth noting that the SEER database may not comprehensively encompass all cases of HB‐NENs, especially those pertaining to diminutive tumors that may exhibit benign characteristics and consequently evade classification as malignant neoplasms. For instance, data concerning numerous diminutive and seemingly benign tumors, such as hepatic lesions, may elude inclusion within the SEER database. Consequently, it is plausible that we might inadvertently underestimate the actual incidence and prevalence rates of HB‐NENs. Furthermore, it is imperative to acknowledge that the SEER database lacks crucial data pertaining to the Ki‐67 and mitotic index, which hold significant relevance in tumor grading [24]. Therefore, the classification of tumors within our study did not incorporate these essential factors, which indicates that conducting expansive, large‐scale investigations in the future is imperative to elucidate and address this specific issue. Finally, in past decades, other treatment options for HB‐NENs mainly including transcatheter arterial chemoembolization, radiofrequency ablation, percutaneous ethanol injection therapy, liver transplantation, novel targeted therapy, and somatostatin analogs [25, 26], have been used to treat patients with locally advanced or distant metastatic HB‐NENs and have had promising survival benefits in selected cohorts [27]. The results of survival analysis might be confounded by the lack of this information.

However, our study also has several strengths. To our knowledge, this study is one of the largest and most novel explorations of HB‐NENs, and long‐term follow‐up data have largely made up for the limitations and encompassed comprehensive epidemiologic and survival information and constructed the nomogram for HB‐NENs.

## 6 Conclusions

In this research, the incidence and prevalence of HB‐NENs have exhibited a consistent increase for nearly 20 years, particularly at specific sites such as the extrahepatic bile duct and gallbladder. Disparities in survival rates were observed based on tumor type, grade, and tumor stage. However, as diagnostic and therapeutic modalities advanced, overall outcomes displayed noticeable improvement. Moreover, a novel nomogram, was developed and validated in the current study. This nomogram demonstrates its efficacy in predicting the 6‐month, 1‐year, and 2‐year survival rates for HB‐NEN patients. It stands poised to furnish physicians and patients with precise and invaluable insights, thereby guiding the treatment strategy for individuals afflicted with HB‐NENs.

## Author Contributions

**Xinlei Zhou:** writing – original draft, data curation, formal analysis. **Chenxi Zhou:** writing – original draft, investigation. **Yi Lu:** validation, formal analysis, investigation. **Shenghao Lin:** software. **Che Fu:** visualization. **Mengfan Wei:** validation. **Leitao Sun:** funding acquisition, methodology. **Kaibo Guo:** conceptualization, funding acquisition, writing – review and editing.

## Funding

This work was supported by the Natural Science Foundation of Zhejiang Province (ZCLQ24H2901), the National Natural Science Foundation of China (82405039), the Medical and Health Technology Program of Zhejiang Province (2024KY1328), the Traditional Chinese Medicine Science and Technology Program of Zhejiang Province (2024ZL712), the Science and Technology Innovation Program for University Students in Zhejiang Province (New Seedling Talent Program) (2024R410A019), and the Science and Technology Innovation Program for University Students in Zhejiang Province (New Seedling Talent Program) (2025R410A001).

## Ethics Statement

The authors have nothing to report.

## Consent

The authors have nothing to report.

## Conflicts of Interest

The authors declare no conflicts of interest.

## Supporting information


**Figure S1:** Analytical cohort and exclusion criteria of patients with hepatobiliary neuroendocrine neoplasms (HB‐NENs) for studying the prognositc factors. ICD‐O‐3, International Classification of Disease for Oncology, Third Edition; SEER, the surveillance, epidemiology, and end results.

**Figure S2:** Hepatobiliary neuroendocrine neoplasms (HB‐NENs) incidence trends by tumor site and tumor type. (A) Annual age‐adjusted incidence of all HB‐NENs by tumor site (1975–2020) including liver, gallbladder, and extrahepatic bile duct. (B) Time‐trend analyzes of the incidence of HB‐NENs by tumor type (1975–2020), including neuroendocrine tumors (NET) and neuroendocrine carcinomas (NEC).

**Figure S3:** The calibration of the nomogram using the validation set and the decision curve analysis of the nomogram predicting 6‐month, 1‐year and 2‐year survival probabilities. (A–C) Calibration plots of the nomogram for 6‐month, 1‐year and 2‐year survival probabilities in the validation set. The gray line represents the ideal nomogram, and the orange line represents the observed nomogram. The predicted probability of OS by the nomogram is projected onto the x‐axis, and the actual OS is projected onto the *y*‐axis. Error bars indicate 95% CIs. (D–F) The decision curve of the nomogram predicting 6‐month, 1‐year and 2‐year survival probabilities was plotted. The *x*‐axis represents the threshold probability and the *y*‐axis represents the net benefit. The bold line represents one extreme situation that no patients suffered death, the solid line represents that all patients experience death, and the dashed line reveals the net benefit of the nomogram.

**Table S1:** Histological ICD codes used to identify NEN patients from SEER.
**Table S2:** Incidence of hepatobiliary neuroendocrine neoplasms (HB‐NENs) over time rates are per 1 000 000 and age‐adjusted to the 2000 US Std population.
**Table S3:** Incidence of liver neuroendocrine neoplasms (LNENs) over time rates are per 1 000 000 and age‐adjusted to the 2000 US Std population.
**Table S4:** Incidence of gallbladder neuroendocrine neoplasms (GNENs) over time rates are per 1 000 000 and age‐adjusted to the 2000 US Std population.
**Table S5:** Incidence of extrahepatic bile duct neuroendocrine neoplasms (ENENs) over time rates are per 1 000 000 and age‐adjusted to the 2000 US Std population.
**Table S6:** Incidence of neuroendocrine tumors (NETs) over time rates are per 1 000 000 and age‐adjusted to the 2000 US Std population.
**Table S7:** Incidence of neuroendocrine carcinomas (NECs) over time rates are per 1 000 000 and age‐adjusted to the 2000 US Std population.
**Table S8:** Nomogram points of the prognostic factors for OS.
**Table S9:** Total points of prognostic factors for predicting 6‐month survival probability.
**Table S10:** Total points of prognostic factors for predicting 1‐year survival probability.
**Table S11:** Total points of prognostic factors for predicting 2‐year survival probability.

## Data Availability

All data were extracted from The Surveillance, Epidemiology, and End Results (SEER) database (https://seer.cancer.gov/).
