# Supplementary figures and images for: Epidemiologic Trends and Factors Associated With Overall Survival for Patients With Hepatobiliary Neuroendocrine Neoplasms in the United States
Source: Cancer Rep (Hoboken). 2025 Nov 28;8(12):e70410. doi: 10.1002/cnr2.70410 (PMC12662706; doi:10.1002/cnr2.70410)

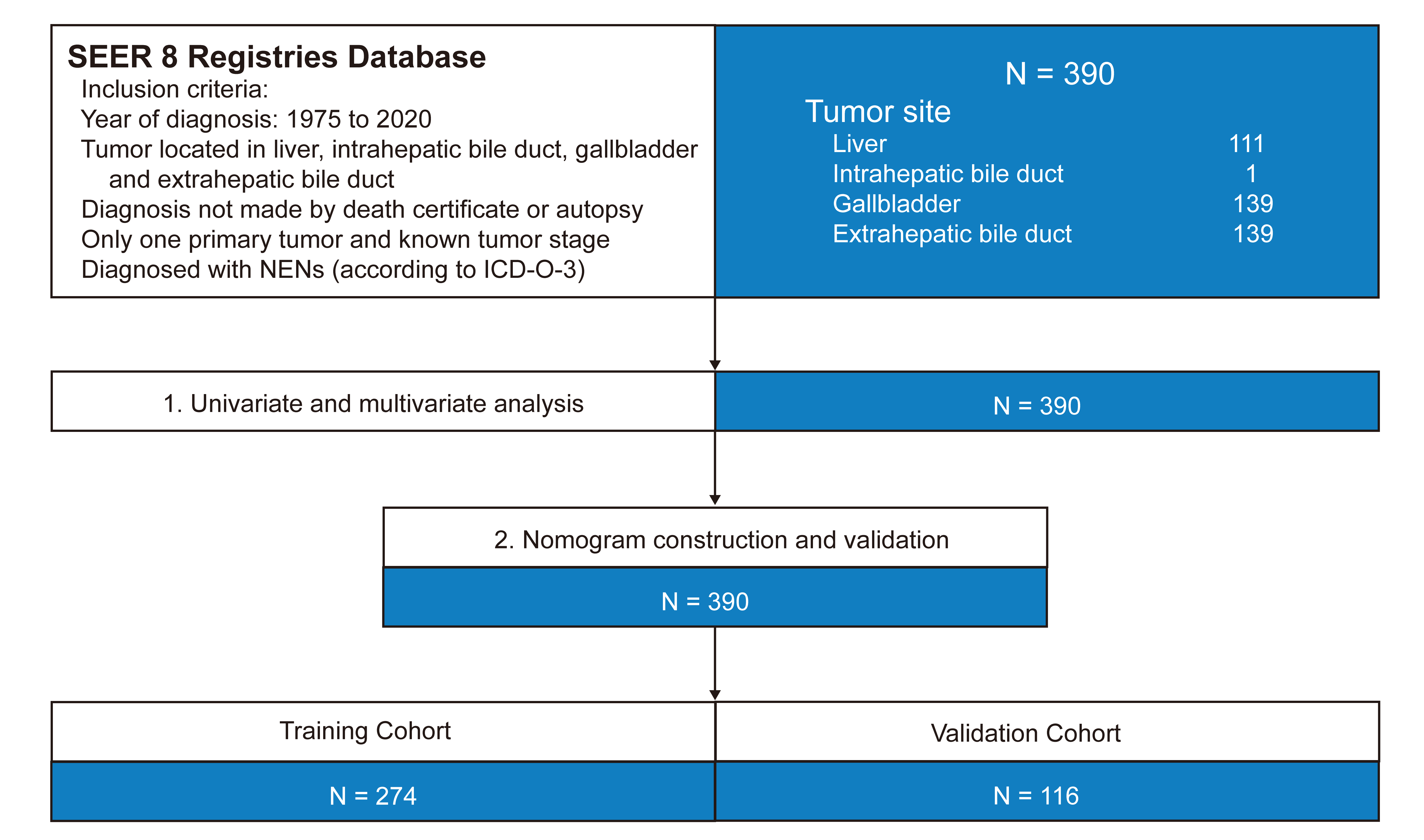

Supplement: Supplementary file 1 — Figure S1: Analytical cohort and exclusion criteria of patients with hepatobiliary neuroendocrine neoplasms (HB‐NENs) for studying the prognositc factors. ICD‐O‐3, International Classification of Disease for Oncology, Third Edition; SEER, the surveillance, epidemiology, and end results. [file CNR2-8-e70410-s002.tif]

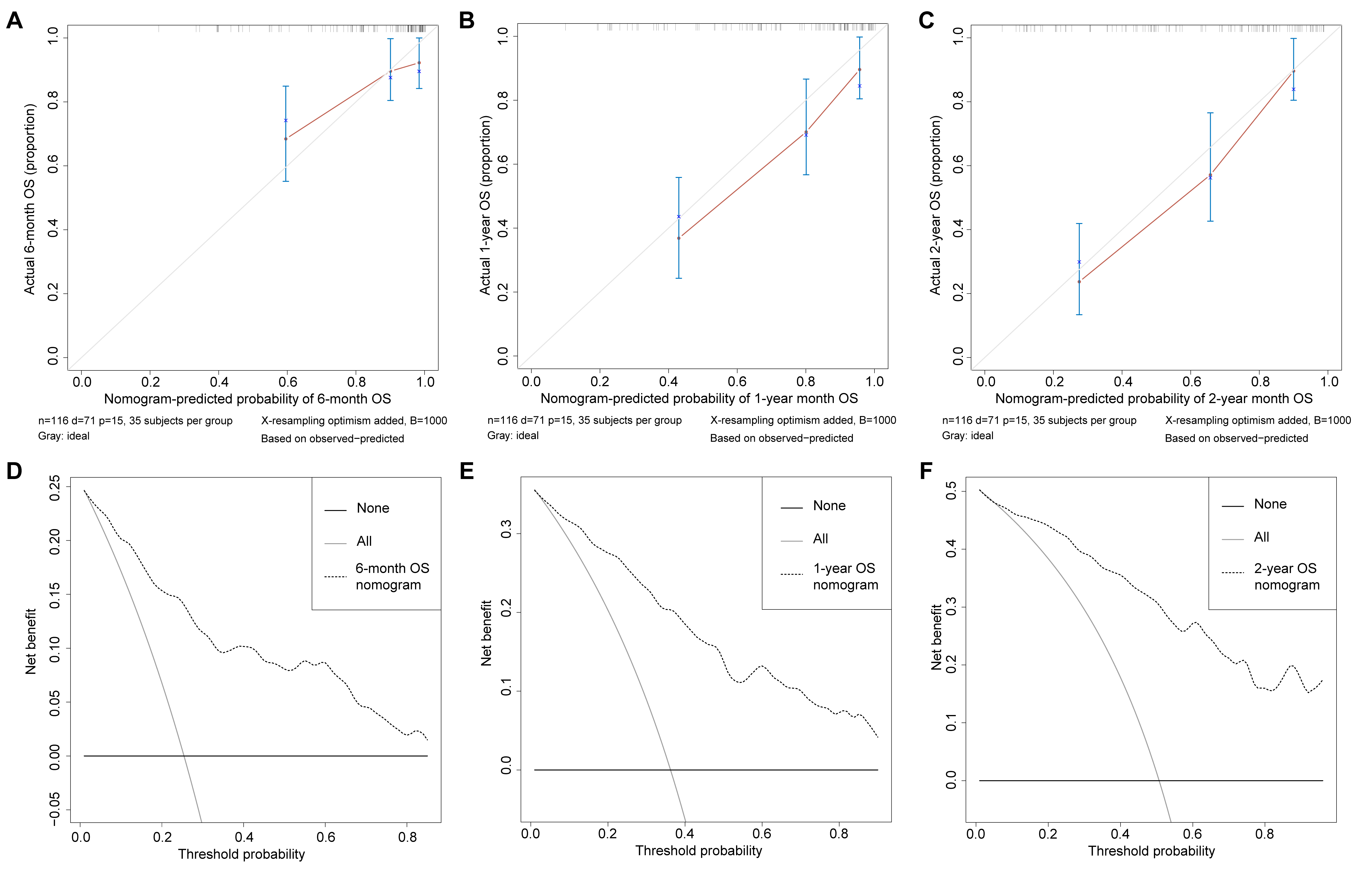

Supplement: Supplementary file 3 — Figure S3: The calibration of the nomogram using the validation set and the decision curve analysis of the nomogram predicting 6‐month, 1‐year and 2‐year survival probabilities. (A–C) Calibration plots of the nomogram for 6‐month, 1‐year and 2‐year survival probabilities in the validation set. The gray line represents the ideal nomogram, and the orange line represents the observed nomogram. The predicted probability of OS by the nomogram is projected onto the x‐axis, and the actual OS is projected onto the y‐axis. Error bars indicate 95% CIs. (D–F) The decision curve of the nomogram predicting 6‐month, 1‐year and 2‐year survival probabilities was plotted. The x‐axis represents the threshold probability and the y‐axis represents the net benefit. The bold line represents one extreme situation that no patients suffered death, the solid line represents that all patients experience death, and the dashed line reveals the net benefit of the nomogram. [file CNR2-8-e70410-s004.tif]
