# Supplementary material for: Epidemiologic Trends and Factors Associated With Overall Survival for Patients With Hepatobiliary Neuroendocrine Neoplasms in the United States
Source: Cancer Rep (Hoboken). 2025 Nov 28;8(12):e70410. doi: 10.1002/cnr2.70410 (PMC12662706; doi:10.1002/cnr2.70410)
Supplement: Supplementary file 4 — Table S1: Histological ICD codes used to identify NEN patients from SEER. Table S2: Incidence of hepatobiliary neuroendocrine neoplasms (HB‐NENs) over time rates are per 1 000 000 and age‐adjusted to the 2000 US Std population. Table S3: Incidence of liver neuroendocrine neoplasms (LNENs) over time rates are per 1 000 000 and age‐adjusted to the 2000 US Std population. Table S4: Incidence of gallbladder neuroendocrine neoplasms (GNENs) over time rates are per 1 000 000 and age‐adjusted to the 2000 US Std population. Table S5: Incidence of extrahepatic bile duct neuroendocrine neoplasms (ENENs) over time rates are per 1 000 000 and age‐adjusted to the 2000 US Std population. Table S6: Incidence of neuroendocrine tumors (NETs) over time rates are per 1 000 000 and age‐adjusted to the 2000 US Std population. Table S7: Incidence of neuroendocrine carcinomas (NECs) over time rates are per 1 000 000 and age‐adjusted to the 2000 US Std population. Table S8: Nomogram points of the prognostic factors for OS. Table S9: Total points of prognostic factors for predicting 6‐month survival probability. Table S10: Total points of prognostic factors for predicting 1‐year survival probability. Table S11: Total points of prognostic factors for predicting 2‐year survival probability. [file CNR2-8-e70410-s001.docx]

Supplementary Table 1. Histological ICD codes used to identify NEN patients from SEER

| **NEN Histology** | **ICD Code** |
| --- | --- |
| **NET Histology** |  |
| Carcinoid | 8240/3 |
| Atypical carcinoid tumor | 8249/3 |
| **NEC Histology** |  |
| Neuroendocrine carcinoma | 8246/3 |
| Large cell neuroendocrine carcinoma | 8103/3 |
| Small cell neuroendocrine carcinoma | 8041/3 |
| Mixed islet cell/exocrine adenocarcinoma | 8154/3 |

Supplementary Table 2. Incidence of hepatobiliary neuroendocrine neoplasms (HB-NENs) over time

| **Year of diagnosis** | **Age-Adjusted Rate/Trend** | **Standard Error** | **Lower Confidence Interval** | **Upper Confidence Interval** |
| --- | --- | --- | --- | --- |
| 1992 | 0.74 | 0.17 | 0.45 | 1.14 |
| 1993 | 0.38 | 0.12 | 0.19 | 0.69 |
| 1994 | 0.29 | 0.10 | 0.13 | 0.55 |
| 1995 | 0.76 | 0.16 | 0.48 | 1.15 |
| 1996 | 0.74 | 0.16 | 0.46 | 1.12 |
| 1997 | 0.68 | 0.15 | 0.41 | 1.04 |
| 1998 | 0.79 | 0.16 | 0.50 | 1.17 |
| 1999 | 0.91 | 0.17 | 0.60 | 1.31 |
| 2000 | 0.76 | 0.10 | 0.57 | 0.99 |
| 2001 | 0.87 | 0.11 | 0.67 | 1.12 |
| 2002 | 0.84 | 0.11 | 0.64 | 1.08 |
| 2003 | 0.87 | 0.11 | 0.67 | 1.11 |
| 2004 | 0.90 | 0.11 | 0.69 | 1.14 |
| 2005 | 1.10 | 0.12 | 0.87 | 1.36 |
| 2006 | 1.02 | 0.12 | 0.81 | 1.28 |
| 2007 | 1.00 | 0.11 | 0.79 | 1.24 |
| 2008 | 1.03 | 0.11 | 0.82 | 1.28 |
| 2009 | 0.71 | 0.10 | 0.54 | 0.93 |
| 2010 | 1.19 | 0.12 | 0.97 | 1.45 |
| 2011 | 1.04 | 0.11 | 0.83 | 1.29 |
| 2012 | 1.05 | 0.11 | 0.84 | 1.29 |
| 2013 | 1.03 | 0.11 | 0.82 | 1.27 |
| 2014 | 1.29 | 0.12 | 1.06 | 1.55 |
| 2015 | 0.98 | 0.11 | 0.78 | 1.21 |
| 2016 | 1.08 | 0.11 | 0.88 | 1.32 |
| 2017 | 1.24 | 0.12 | 1.02 | 1.49 |
| 2018 | 1.22 | 0.11 | 1.01 | 1.47 |
| 2019 | 1.31 | 0.12 | 1.09 | 1.56 |
| 2020 | 1.30 | 0.11 | 1.09 | 1.54 |

Rates are per 1000000 and age-ajusted to the 2000 US Std Population.

Supplementary Table 3. Incidence of liver neuroendocrine neoplasms (LNENs) over time

| **Year of diagnosis** | **Age-Adjusted Rate/Trend** | **Standard Error** | **Lower Confidence Interval** | **Upper Confidence Interval** |
| --- | --- | --- | --- | --- |
| 1992 | 0.35 | 0.12 | 0.16 | 0.65 |
| 1993 | 0.21 | 0.09 | 0.08 | 0.45 |
| 1994 | 0.10 | 0.06 | 0.02 | 0.28 |
| 1995 | 0.50 | 0.13 | 0.28 | 0.84 |
| 1996 | 0.28 | 0.10 | 0.12 | 0.55 |
| 1997 | 0.20 | 0.08 | 0.07 | 0.43 |
| 1998 | 0.45 | 0.12 | 0.25 | 0.76 |
| 1999 | 0.36 | 0.11 | 0.18 | 0.65 |
| 2000 | 0.43 | 0.08 | 0.29 | 0.61 |
| 2001 | 0.50 | 0.08 | 0.35 | 0.69 |
| 2002 | 0.35 | 0.07 | 0.22 | 0.51 |
| 2003 | 0.42 | 0.08 | 0.28 | 0.59 |
| 2004 | 0.39 | 0.07 | 0.26 | 0.57 |
| 2005 | 0.46 | 0.08 | 0.32 | 0.64 |
| 2006 | 0.55 | 0.08 | 0.40 | 0.74 |
| 2007 | 0.37 | 0.07 | 0.25 | 0.54 |
| 2008 | 0.42 | 0.07 | 0.29 | 0.59 |
| 2009 | 0.24 | 0.05 | 0.15 | 0.37 |
| 2010 | 0.50 | 0.08 | 0.36 | 0.68 |
| 2011 | 0.50 | 0.08 | 0.36 | 0.68 |
| 2012 | 0.46 | 0.07 | 0.33 | 0.63 |
| 2013 | 0.36 | 0.07 | 0.24 | 0.51 |
| 2014 | 0.47 | 0.07 | 0.34 | 0.64 |
| 2015 | 0.30 | 0.06 | 0.20 | 0.44 |
| 2016 | 0.35 | 0.06 | 0.24 | 0.49 |
| 2017 | 0.49 | 0.07 | 0.36 | 0.66 |
| 2018 | 0.41 | 0.07 | 0.29 | 0.57 |
| 2019 | 0.42 | 0.07 | 0.30 | 0.58 |
| 2020 | 0.49 | 0.07 | 0.36 | 0.65 |

Rates are per 1000000 and age-ajusted to the 2000 US Std Population.

Supplementary Table 4. Incidence of gallbladder neuroendocrine [neoplasm](javascript:;)s (GNENs) over time

| **Year of diagnosis** | **Age-Adjusted Rate/Trend** | **Standard Error** | **Lower Confidence Interval** | **Upper Confidence Interval** |
| --- | --- | --- | --- | --- |
| 1992 | 0.18 | 0.08 | 0.06 | 0.41 |
| 1993 | 0.07 | 0.05 | 0.01 | 0.26 |
| 1994 | 0.13 | 0.06 | 0.03 | 0.33 |
| 1995 | 0.13 | 0.07 | 0.03 | 0.33 |
| 1996 | 0.14 | 0.07 | 0.04 | 0.35 |
| 1997 | 0.24 | 0.09 | 0.10 | 0.49 |
| 1998 | 0.20 | 0.08 | 0.07 | 0.43 |
| 1999 | 0.25 | 0.09 | 0.11 | 0.50 |
| 2000 | 0.19 | 0.05 | 0.10 | 0.32 |
| 2001 | 0.19 | 0.05 | 0.10 | 0.32 |
| 2002 | 0.22 | 0.06 | 0.13 | 0.36 |
| 2003 | 0.27 | 0.06 | 0.17 | 0.42 |
| 2004 | 0.25 | 0.06 | 0.15 | 0.40 |
| 2005 | 0.29 | 0.06 | 0.18 | 0.44 |
| 2006 | 0.27 | 0.06 | 0.17 | 0.42 |
| 2007 | 0.31 | 0.06 | 0.21 | 0.46 |
| 2008 | 0.35 | 0.07 | 0.23 | 0.52 |
| 2009 | 0.24 | 0.06 | 0.14 | 0.38 |
| 2010 | 0.32 | 0.06 | 0.21 | 0.47 |
| 2011 | 0.27 | 0.06 | 0.17 | 0.41 |
| 2012 | 0.27 | 0.06 | 0.17 | 0.41 |
| 2013 | 0.27 | 0.05 | 0.17 | 0.40 |
| 2014 | 0.42 | 0.07 | 0.30 | 0.58 |
| 2015 | 0.38 | 0.07 | 0.26 | 0.54 |
| 2016 | 0.24 | 0.05 | 0.15 | 0.36 |
| 2017 | 0.33 | 0.06 | 0.22 | 0.47 |
| 2018 | 0.35 | 0.06 | 0.25 | 0.50 |
| 2019 | 0.38 | 0.06 | 0.27 | 0.53 |
| 2020 | 0.33 | 0.06 | 0.23 | 0.47 |

Rates are per 1000000 and age-ajusted to the 2000 US Std Population.

Supplementary Table 5. Incidence of extrahepatic bile duct neuroendocrine [neoplasm](javascript:;)s (ENENs) over time

| **Year of diagnosis** | **Age-Adjusted Rate/Trend** | **Standard Error** | **Lower Confidence Interval** | **Upper Confidence Interval** |
| --- | --- | --- | --- | --- |
| 1992 | 0.22 | 0.09 | 0.08 | 0.47 |
| 1993 | 0.10 | 0.06 | 0.02 | 0.30 |
| 1994 | 0.07 | 0.05 | 0.01 | 0.25 |
| 1995 | 0.13 | 0.06 | 0.04 | 0.33 |
| 1996 | 0.32 | 0.11 | 0.14 | 0.59 |
| 1997 | 0.24 | 0.09 | 0.10 | 0.48 |
| 1998 | 0.13 | 0.07 | 0.04 | 0.34 |
| 1999 | 0.29 | 0.10 | 0.13 | 0.55 |
| 2000 | 0.14 | 0.04 | 0.07 | 0.26 |
| 2001 | 0.18 | 0.05 | 0.10 | 0.31 |
| 2002 | 0.27 | 0.06 | 0.16 | 0.42 |
| 2003 | 0.18 | 0.05 | 0.09 | 0.30 |
| 2004 | 0.25 | 0.06 | 0.15 | 0.39 |
| 2005 | 0.35 | 0.07 | 0.23 | 0.51 |
| 2006 | 0.20 | 0.05 | 0.11 | 0.33 |
| 2007 | 0.31 | 0.06 | 0.20 | 0.45 |
| 2008 | 0.26 | 0.06 | 0.16 | 0.39 |
| 2009 | 0.23 | 0.05 | 0.14 | 0.37 |
| 2010 | 0.37 | 0.07 | 0.25 | 0.52 |
| 2011 | 0.27 | 0.06 | 0.17 | 0.41 |
| 2012 | 0.32 | 0.06 | 0.21 | 0.46 |
| 2013 | 0.40 | 0.07 | 0.28 | 0.56 |
| 2014 | 0.40 | 0.07 | 0.28 | 0.55 |
| 2015 | 0.29 | 0.06 | 0.19 | 0.43 |
| 2016 | 0.50 | 0.07 | 0.36 | 0.67 |
| 2017 | 0.42 | 0.07 | 0.29 | 0.58 |
| 2018 | 0.46 | 0.07 | 0.33 | 0.61 |
| 2019 | 0.50 | 0.07 | 0.37 | 0.67 |
| 2020 | 0.48 | 0.07 | 0.35 | 0.63 |

Rates are per 1000000 and age-ajusted to the 2000 US Std Population.

Supplementary Table 6. Incidence of neuroendocrine tumors (NETs) over time

| **Year of diagnosis** | **Age-Adjusted Rate/Trend** | **Standard Error** | **Lower Confidence Interval** | **Upper Confidence Interval** |
| --- | --- | --- | --- | --- |
| 1992 | 0.33 | 0.11 | 0.15 | 0.62 |
| 1993 | 0.20 | 0.08 | 0.07 | 0.44 |
| 1994 | 0.26 | 0.09 | 0.11 | 0.52 |
| 1995 | 0.49 | 0.13 | 0.27 | 0.83 |
| 1996 | 0.31 | 0.10 | 0.14 | 0.59 |
| 1997 | 0.41 | 0.12 | 0.21 | 0.71 |
| 1998 | 0.32 | 0.10 | 0.15 | 0.59 |
| 1999 | 0.35 | 0.11 | 0.18 | 0.63 |
| 2000 | 0.40 | 0.08 | 0.27 | 0.58 |
| 2001 | 0.43 | 0.08 | 0.29 | 0.61 |
| 2002 | 0.38 | 0.07 | 0.25 | 0.55 |
| 2003 | 0.30 | 0.06 | 0.19 | 0.45 |
| 2004 | 0.35 | 0.07 | 0.23 | 0.51 |
| 2005 | 0.54 | 0.09 | 0.39 | 0.74 |
| 2006 | 0.42 | 0.07 | 0.29 | 0.60 |
| 2007 | 0.37 | 0.07 | 0.25 | 0.53 |
| 2008 | 0.29 | 0.06 | 0.18 | 0.43 |
| 2009 | 0.36 | 0.07 | 0.24 | 0.52 |
| 2010 | 0.33 | 0.06 | 0.22 | 0.47 |
| 2011 | 0.29 | 0.06 | 0.19 | 0.44 |
| 2012 | 0.25 | 0.05 | 0.16 | 0.38 |
| 2013 | 0.20 | 0.05 | 0.12 | 0.32 |
| 2014 | 0.54 | 0.08 | 0.39 | 0.71 |
| 2015 | 0.40 | 0.07 | 0.27 | 0.55 |
| 2016 | 0.47 | 0.07 | 0.34 | 0.63 |
| 2017 | 0.59 | 0.08 | 0.44 | 0.77 |
| 2018 | 0.47 | 0.07 | 0.34 | 0.64 |
| 2019 | 0.57 | 0.08 | 0.43 | 0.75 |
| 2020 | 0.59 | 0.08 | 0.45 | 0.77 |

Rates are per 1000000 and age-ajusted to the 2000 US Std Population.

Supplementary Table 7.Incidence of neuroendocrine carcinomas (NECs) over time

| **Year of diagnosis** | **Age-Adjusted Rate/Trend** | **Standard Error** | **Lower Confidence Interval** | **Upper Confidence Interval** |
| --- | --- | --- | --- | --- |
| 1992 | 0.41 | 0.12 | 0.20 | 0.72 |
| 1993 | 0.18 | 0.08 | 0.06 | 0.42 |
| 1994 | 0.03 | 0.03 | 0.00 | 0.17 |
| 1995 | 0.27 | 0.10 | 0.11 | 0.53 |
| 1996 | 0.42 | 0.12 | 0.22 | 0.73 |
| 1997 | 0.27 | 0.10 | 0.12 | 0.53 |
| 1998 | 0.46 | 0.12 | 0.25 | 0.78 |
| 1999 | 0.55 | 0.13 | 0.32 | 0.88 |
| 2000 | 0.36 | 0.07 | 0.23 | 0.53 |
| 2001 | 0.44 | 0.08 | 0.30 | 0.63 |
| 2002 | 0.46 | 0.08 | 0.32 | 0.65 |
| 2003 | 0.57 | 0.09 | 0.41 | 0.77 |
| 2004 | 0.55 | 0.09 | 0.39 | 0.74 |
| 2005 | 0.55 | 0.09 | 0.40 | 0.75 |
| 2006 | 0.60 | 0.09 | 0.44 | 0.80 |
| 2007 | 0.62 | 0.09 | 0.46 | 0.82 |
| 2008 | 0.74 | 0.10 | 0.57 | 0.96 |
| 2009 | 0.35 | 0.07 | 0.23 | 0.51 |
| 2010 | 0.86 | 0.10 | 0.67 | 1.09 |
| 2011 | 0.75 | 0.10 | 0.57 | 0.96 |
| 2012 | 0.80 | 0.10 | 0.62 | 1.02 |
| 2013 | 0.83 | 0.10 | 0.65 | 1.05 |
| 2014 | 0.75 | 0.09 | 0.58 | 0.95 |
| 2015 | 0.58 | 0.08 | 0.43 | 0.77 |
| 2016 | 0.62 | 0.08 | 0.47 | 0.80 |
| 2017 | 0.65 | 0.08 | 0.49 | 0.83 |
| 2018 | 0.75 | 0.09 | 0.59 | 0.94 |
| 2019 | 0.74 | 0.09 | 0.58 | 0.93 |
| 2020 | 0.71 | 0.08 | 0.55 | 0.89 |

Rates are per 1000000 and age-ajusted to the 2000 US Std Population.

Supplementary Table 8. Nomogram points of the prognostic factors for OS

| **Characteristics** | **Score** |
| --- | --- |
| **Age at diagnosis** |  |
| <60 year | 0 |
| 60-74 year | 29 |
| ≥75 year | 78 |
| **Race** |  |
| White | 22 |
| Black | 0 |
| Other | 59 |
| **Tumor size** |  |
| ≤20 mm | 0 |
| 21-50 mm | 40 |
| >50 mm | 47 |
| Unknown | 54 |
| **Grade** |  |
| I-II | 0 |
| III-IV | 80 |
| Unknown | 16 |
| **Tumor type** |  |
| NEC | 83 |
| NET | 0 |
| **Tumor stage** |  |
| Distant | 77 |
| Localized | 0 |
| Regional | 63 |
| **Chemotherapy** |  |
| No | 20 |
| Yes | 0 |
| **Surgery** |  |
| No | 100 |
| Yes | 0 |

Supplementary Table 9. Total points of prognostic factors for predicting 6-month survival probability

| **Total points** | **6-month survival probability** |
| --- | --- |
| 187 | 0.95 |
| 244 | 0.9 |
| 277 | 0.85 |
| 302 | 0.80 |
| 339 | 0.70 |
| 367 | 0.6 |
| 391 | 0.5 |
| 413 | 0.4 |
| 434 | 0.3 |
| 457 | 0.2 |
| 485 | 0.1 |
| 506 | 0.05 |

Supplementary Table 10. Total points of prognostic factors for predicting 1-year survival probability

| **Total points** | **1-year survival probability** |
| --- | --- |
| 139 | 0.95 |
| 196 | 0.9 |
| 229 | 0.85 |
| 254 | 0.80 |
| 291 | 0.70 |
| 319 | 0.6 |
| 343 | 0.5 |
| 365 | 0.4 |
| 386 | 0.3 |
| 409 | 0.2 |
| 437 | 0.1 |
| 458 | 0.05 |

Supplementary Table 11. Total points of prognostic factors for predicting 2-year survival probability

| **Total points** | **2-year survival probability** |
| --- | --- |
| 81 | 0.95 |
| 137 | 0.9 |
| 171 | 0.85 |
| 196 | 0.80 |
| 233 | 0.70 |
| 261 | 0.6 |
| 285 | 0.5 |
| 307 | 0.4 |
| 328 | 0.3 |
| 351 | 0.2 |
| 379 | 0.1 |
| 399 | 0.05 |
